# Effect of foot orthoses on lower extremity kinetics during running: a systematic literature review

**DOI:** 10.1186/1757-1146-1-13

**Published:** 2008-11-17

**Authors:** Andrew McMillan, Craig Payne

**Affiliations:** 1 Department of Podiatry, La Trobe University, Bundoora, Vic. 3086, Australia

## Abstract

**Background:**

Throughout the period of one year, approximately 50% of recreational runners will sustain an injury that disrupts their training regimen. Foot orthoses have been shown to be clinically effective in the prevention and treatment of several running-related conditions, yet the physical effect of this intervention during running remains poorly understood. The aim of this literature review was therefore to evaluate the effect of foot orthoses on lower extremity forces and pressure (kinetics) during running.

**Methods:**

A systematic search of electronic databases including Medline (1966-present), CINAHL, SportDiscus, and The Cochrane Library occurred on 7 May 2008. Eligible articles were selected according to pre-determined criteria. Methodological quality was evaluated by use of the Quality Index as described by Downs & Black, followed by critical analysis according to outcome variables.

**Results:**

The most widely reported kinetic outcomes were loading rate and impact force, however the effect of foot orthoses on these variables remains unclear. In contrast, current evidence suggests that a reduction in the rearfoot inversion moment is the most consistent kinetic effect of foot orthoses during running.

**Conclusion:**

The findings of this review demonstrate systematic effects that may inform the direction of future research, as further evidence is required to define the mechanism of action of foot orthoses during running. Continuation of research in this field will enable targeting of design parameters towards biomechanical variables that are supported by evidence, and may lead to advancements in clinical efficacy.

## Background

Throughout the period of one year, approximately 50% of recreational runners will sustain an injury that disrupts their training regimen [1,2]. Intrinsic risk factors shown to consistently correlate with running injury include previous injury [3-7] and limited running experience [3,8-10]. Support for an association between foot morphology and specific running-related injuries has also been shown in several clinical studies. For example, the association between pes cavus and lower extremity stress fracture is well supported [7,11-15], while further evidence demonstrates an association between pes planus and medial tibial stress syndrome [11,13,16-18]. An association between foot posture and plantar fasciitis in the running population is less convincing [7,19-21], however Irving et al. [22] recently found pronated foot alignment to be a risk factor for this condition. An association between foot pronation and patellofemoral pain has also been suggested in the literature [7,11,23,24], however this relationship has been contested by several prospective and cross-sectional cohort studies [25-30].

Patellofemoral pain syndrome is reported to be the most commonly encountered running-related injury [1,31,32], accounting for between 11% and 16% of conditions [31,32]. The incidence of stress fracture has been found to vary from 4% to 15% of running injuries, with the tibia, navicular, and femur being the most common sites [31-33]. Medial tibial stress syndrome and plantar fasciitis have been found to have similar rates of incidence, accounting for between 4% and 8% of running-related injuries [31,32].

The clinical effectiveness of foot orthoses has been demonstrated in clinical trials for either the prevention [34-37] or treatment [38-43] of the running-related conditions described above. However at the time of writing, no systematic review evaluating the mechanism of action of foot orthoses during running had been published. This limitation has consequences in relation to dispensing foot orthoses, as without an understanding of the intervention effect, the presumed action may not be produced as intended.

Several literature reviews have evaluated the effects of foot orthoses on lower extremity position and movement (kinematics) without a systematic search strategy [44-47]. These reviews have described the effect of foot orthoses on kinematic variables to be small and non-systematic. As a result, research into the biomechanics of foot orthoses has increasingly focussed on lower extremity force and pressure (kinetics). However, the effect of foot orthoses on kinetic variables during running had not been systematically evaluated at the time of writing. The aim of this literature review was therefore to systematically collect all published research in this topic, and critically evaluate the methodology and experimental findings.

## Methods

A systematic search of electronic databases including Medline (1966-present), CINAHL, SportDiscus, and The Cochrane Library occurred on 7 May 2008. The search terms foot orthotic$, foot orthos$s and insole$ were used in conjunction with the terms kinetic$, biomechanic$, running, and force$ in various combinations (Table 1). The search strategy was limited to articles published in the English language. Targeted searching of relevant journals also occurred following bibliographic review of retrieved articles.

**Table 1:** Search terminology and generated citations according to database title.

Search Term	Medline	CINAHL	SPORT Discuss	Cochrane Library	Total
foot orthotic$ AND kinetic$	5	7	7	2	21
foot orthos$s AND kinetic$	29	50	22	4	105
Insole$ AND kinetic$	9	9	14	1	33

foot orthotic$ AND biomechanic$	17	21	41	13	92
foot orthos$s AND biomechanic$	121	185	94	14	414
Insole$ AND biomechanic$	73	26	112	9	220

foot orthotic$ AND running	7	11	25	16	59
foot orthos$s AND running	15	42	21	13	91
Insole$ AND running	24	15	59	7	105

Orthotic$ AND running AND biomechanic$	35	17	71	7	130
Orthos$s AND running AND biomechanic$	21	36	34	6	97
Insole$ AND running AND biomechanic$	12	6	26	3	47

foot orthotic$ AND force$	3	3	6	18	30
foot orthos$s AND force$	52	73	33	21	179
Insole$ AND force$	67	28	75	8	178

Total	490	529	640	142	1801

Articles accepted for inclusion were required to be published in peer-reviewed journals, and report the findings of original experimental or quasi-experimental research. Articles were excluded according to the following criteria:

• Measurement of effects during walking

• Kinematic variables exclusively investigated.

• Orthoses other than foot orthoses exclusively investigated.

Electromyographic (EMG) studies were also excluded, as they measured biophysical variables that are distinct from kinetics. Furthermore, in accordance with definitions contained in the Australian Podiatry Council's 'Guidelines on Orthotic Therapy' [48], studies investigating the effects of insoles limited to cushioning properties were also excluded. Titles and abstracts of all citations generated by the search were assessed by one author according to the inclusion and exclusion criteria above, with articles printed in full-text as required.

All articles accepted for review underwent methodological assessment to evaluate the research quality. This process occurred in accordance with the Quality Index described by Downs & Black [49], in which a systematic checklist is used to evaluate the external and internal validities of clinical trials. This checklist was adjusted to exclude 12 questions deemed to be less relevant to the articles assessed in this review, resulting in the retention of 15 questions (Table 2). Additionally, the protocol and specific design features of each study were extracted, with particular focus on condition randomisation, participant running experience, condition acclimatisation and orthosis design.

**Table 2:** Quality assessment of included articles (adapted from Downs & Black [49])

	Reference:	[51]	[52]	[58]	[53]	[54]	[60]	[55]	[59]	[56]	[57]
1	Is the hypothesis/aim of the study clearly described ?	1	1	1	1	1	1	1	1	1	1

2	Are the main outcomes to be measured clearly described in the Introduction or Methods section ?	1	1	1	1	1	1	1	1	1	1

3	Are the characteristics of the patients included in the study clearly described ?	0	0	0	0	1	1	1	0	0	0

4	Are the interventions of interest clearly described ?	1	1	1	1	1	1	1	1	1	1

5	Are the main findings of the study clearly described ?	1	1	1	1	1	1	1	1	1	1

6	Does the study provide estimates of the random variability in the data for the main outcomes ?	1	1	1	1	1	1	1	1	1	1

7	Have actual probability values been reported (e.g. 0.035 rather than <0.05) for the main outcomes except where the probability value is less than 0.001 ?	1	0	1	1	1	0	1	0	1	1

8	Were the subjects asked to participate in the study representative of the entire population from which they were recruited ?	0	0	1	0	0	0	0	0	1	0

9	Were those subjects who were prepared to participate representative of the entire population from which they were recruited ?	0	0	0	1	0	0	0	0	1	0

10	Was an attempt made to blind study subjects to the intervention they have received ?	0	0	0	0	0	0	0	0	0	0

11	Was an attempt made to blind those measuring the main outcomes of the intervention ?	0	0	0	0	0	0	0	0	0	0

12	If any of the results of the study were based on "data dredging", was this made clear ?	1	1	1	1	1	0	1	1	1	1

13	Were the statistical tests used to assess the main outcomes appropriate ?	1	1	1	1	1	0	1	1	1	1

14	Were the main outcome measures used accurate (valid and reliable) ?	1	1	1	1	1	0	1	1	1	1

15	Were study subjects randomised to intervention groups ?	1	0	0	1	0	0	1	1	0	1

	Quality Index Score (max score = 15)	10	8	10	11	10	6	11	9	11	10

	Quality Index %	67	53	67	73	67	40	73	60	73	67

(0 = no/unable to determine, 1 = yes)

Following methodological assessment, articles were grouped and discussed according to kinetic outcome variables. However the evaluation was unable to be conducted as a meta-analysis, due to heterogenicity in experimental designs.

## Results

The search process generated a total 1801 citations for initial screening (Table 1), of which 1770 were excluded on review of title and abstract. 31 articles were printed in full-text for further consideration, of which 10 were eligible for final inclusion (Table 3). All included articles were published between 1991 and 2008, and reported the findings of laboratory-based research with a repeated measures design.

**Table 3:** Articles selected for inclusion

Author	Date	Title	Journal	Ref
Butler et al.	2003	Dual function foot orthosis: effect on shock and control of rearfoot motion.	Foot Ankle Int.	[51]
Dixon	2007	Influence of a commercially available orthotic device on rearfoot eversion and vertical ground reaction force when running in military footwear.	Mil Med.	[52]
Dixon & McNally	2008	Influence of orthotic devices prescribed using pressure data on lower extremity kinematics and pressures beneath the shoe during running.	Clin Biomech.	[58]
Laughton et al.	2003	Effect of strike pattern and orthotic intervention on tibial shock during running.	J Appl Biomech.	[53]
MacLean et al.	2006	Influence of a custom foot orthotic intervention on lower extremity dynamics in healthy runners.	Clin Biomech.	[54]
McPoil & Cornwall	1991	Rigid versus soft foot orthoses: a single subject design.	JAPMA	[60]
Mundermann et al.	2003	Foot orthotics affect lower extremity kinematics and kinetics during running.	Clin Biomech.	[55]
Nigg et al.	2003	Effect of shoe inserts on kinematics, center of pressure, and leg joint moments during running.	Med Sci Sports Exerc.	[59]
Stackhouse et al.	2004	Orthotic intervention in forefoot and rearfoot strike running patterns.	Clin Biomech.	[56]
Williams et al.	2003	Effect of inverted orthoses on lower-extremity mechanics in runners.	Med Sci Sports Exerc.	[57]

The mean Quality Index Score for the articles was 64 % (SD = 10.5), demonstrating limited overall quality (Table 2). The majority of studies demonstrated inadequacy in selecting a representative sample and in the description of participant characteristics, while none attempted to blind subjects or investigators. Four studies included participants without reporting estimates of weekly running mileage. Additionally, several trials failed to randomise the sequence in which conditions occurred, thereby exposing the findings to order effects.

Despite the limitations described above, all studies provided adequate descriptions of outcome variables and orthosis design parameters, and reported findings with estimates of random variability. Furthermore, all used standardised footwear and running speeds during experimental conditions.

### Loading Rate and Peak Impact Force

The vertical loading rate is the vertical impact force quantified with reference to time, and is normally reported as either the maximum or average in Newtons per second (N/s) [50]. Five articles measured this variable by force-plate analysis [51-55].

A study of 8 military recruits [52] found a significant decrease in both the average and peak loading rates while running in prefabricated foot orthoses. Despite subjects in this trial wearing military boots, these findings correspond with two trials [53,55] in which foot orthoses were shown to significantly reduce the loading rates of runners with both normal and excessively pronated foot posture. However, two trials investigating the effects of custom-moulded foot orthoses ([51,54] found no significant effect on loading rates during running.

In addition to investigating loading rates, four of the studies above measured the peak impact force magnitude [52-55]. This variable is the maximum vertical ground-reaction force (GRF) during the initial loading phase of stance [50], and is thereby closely related to the loading rate. This relationship is demonstrated in the findings of these trials, with three studies reporting both variables to be either significantly [52,55] or insignificantly [54] reduced by foot orthoses. In contrast, Laughton et al. [53] found an inverse relationship between these variables (Table 4).

**Table 4:** Quality of articles reporting findings for loading rate and peak impact force.

Ref.	Orthosis Design	Significant effect on loading rate	Significant effect on peak impact force	Quality Index Score (%)	Condition Randomisation	Experienced Runners	Acclimatization Period
[51]	Custom-moulded rigid & soft: 6 degrees rearfoot post	✗	n/a	67	✓	✗	✓

[52]	Prefabricated semi rigid: full length	✓	✓	53	✗	✗	✗

[53]	Custom-moulded semi-rigid: 6 degrees rearfoot post	✓	✗	73	✓	✗	✓

[54]	Custom-moulded semi-rigid: 5 degrees rearfoot post	✗	✗	67	✗	✓	✗

[55]	Custom-moulded semi-rigid: nil post & 6 mm rearfoot/forefoot post	✓	✓	73	✓	✓	✗

Comparison of the foot orthoses used in these trials fails to demonstrate correlations with orthosis design, as conflicting data was collected by use of orthoses with similar features (Table 4). Furthermore, as the Quality Index Scores of the above studies are also very similar, weighting of evidence based on methodological quality would result in equivalent findings (Table 4). An approximately equal proportion of studies with systematic and non-systematic findings failed to ensure that only experienced runners were included, subjects were allocated a period of acclimatisation, and the order of conditions was randomised (Table 4). Furthermore, all trials involved participants running over-ground at very similar speeds, with data collected by comparable equipment.

As the trials described above have similar methodological quality and research designs, current evidence suggests that foot orthoses have non-systematic effects on the loading rate and peak impact force during running.

### Rearfoot Inversion Moment

A resultant joint moment is the rotational force generated at the joint axis by a force applied to a biomechanical lever-arm, and is calculated by multiplying the applied force (Newtons) by the length (metres) of the lever-arm by which it acts [50]. Three articles collected data for the rearfoot inversion moment [54,56,57], all used custom-moulded orthoses with subjects running over a force plate under 3-dimensional video analysis.

All three trials investigating the effect of foot orthoses on rearfoot inversion moments demonstrate a consistent trend. Two of these trials [54,57] report a statistically significant effect, however the trial by Williams et al. [57] compared the effect of orthoses with 4° rearfoot posting to orthoses with 25° posting, finding only the latter to produce a significant effect. However, this discrepancy may be due to the relatively small sample size of this trial (n = 11), as an average 27% decrease in rearfoot inversion moment was measured with the 4° orthosis. These findings are similar to those of the trial that did not reach statistical significance [56], in which a 24% decrease in rearfoot inversion moment was measured, and a post-hoc power calculation revealed that additional subjects were required.

The findings of these studies are consistent despite differences in subject foot morphology, with two trials only including healthy subjects [54,56], and the remaining trial [57] only including subjects with a clinical need for the orthoses. Additionally, while two of these trials were exposed to order effects, the Quality Index Scores are higher than the overall mean for the trials included in this review (Table 5).

**Table 5:** Quality of articles reporting findings for rearfoot inversion moment.

Ref.	Orthosis Design	Quality Index Score (%)	Condition randomisation	Experienced runners	Acclimatization period
[54]	Custom-moulded semi-rigid: 5 degrees rearfoot post	67	✗	✓	✗

[56]	Custom-moulded semi-rigid: 6 degrees rearfoot post	73	✗	✓	✓

[57]	Custom-moulded semi-rigid: 4 degrees & 15–25 degrees rearfoot post	67	✓	✓	✓

These trials suggest that foot orthoses have a systematic effect on the rearfoot inversion moment of runners with both normal and excessively pronated foot posture, and suggest a linear relationship between degree of rearfoot posting and effect magnitude. Furthermore, the findings of these trials contribute significantly to current understanding of the mechanism of action of foot orthoses during running.

### Plantar Pressure

Plantar pressure may be described as the quantity of force acting over the plantar surface area of the foot, and is normally reported as Newtons per centimetre squared (N/cm2) [50]. Two articles [58,59] collected data for plantar pressure during running in custom-moulded orthoses, with conflicting findings.

A trial of 22 runners with recurring lower limb injury found medially-posted foot orthoses to have a consistent effect on plantar pressure during running [58]. This trial used a digital masking technique in which the plantar rearfoot was subdivided into medial and lateral segments, while plantar pressures beneath the metatarsal heads were measured individually. In comparison to control, this trial found a considerable increase in plantar pressure under the lateral surface of the foot with medially-posted orthoses. In contrast, an earlier trial [59] found laterally-posted orthoses to cause deviation of plantar pressure in the same direction, and medially posted orthoses to produce only random effects.

In addition to variations in orthosis design between these two trials (Table 6), there are differences in the technical equipment used, with one trial using a pressure-plate [58] and the other an insole system [59]. This discrepancy may obscure the comparison of results between these two trials, as a pressure-plate measures pressure at the shoe/ground interface, while an insole sensor detects pressure at the foot/orthosis interface.

**Table 6:** Quality of articles reporting findings for plantar pressure.

Ref.	Orthosis Design	Quality Index Score (%)	n =	Condition randomisation	Experienced runners	Acclimatization period
[58]	Custom-moulded EVA with shell: high normal and low arch contour	67	22	✗	✓	✗

[59]	Custom-moulded EVA: 4.5 mm lateral post	60	15	✓	✗	✗

The findings of these trials suggest that plantar pressure beneath the lateral foot may be increased while running in foot orthoses with both medial and lateral posting designs, and that detection of effects may depend on the interface at which pressure is measured. Current evidence therefore suggests that foot orthoses have a variable effect on medio-lateral plantar pressure distribution during running, and that further research into this parameter is required.

### Timing of Peak Impact Force

In addition to reporting the magnitude of peak impact force, one trial [52] measured the timing of peak impact force during running. The findings of this trial demonstrate a systematic delay in the timing of peak impact force with the use of full-length prefabricated foot orthoses. While current evidence for the effect of foot orthoses on this variable is limited to one trial, the findings suggest that further research into the timing of plantar force variables may be productive.

### Force/Time Integral

The force/time integral is also known by the term impulse, and is calculated as the area below the plantar force/time curve [50]. One trial investigated the effects of foot orthoses on the force/time integral during running [60]. While the results of this trial demonstrate a reduction in this variable with custom-moulded orthoses, the single-subject design and poor methodological quality limit the implications of these findings (Table 7). Current evidence for the effect of foot orthoses on the force/time integral during running is therefore insufficient, and unable to suggest systematic changes. Further research is required to investigate this parameter adequately.

**Table 7:** Methodological quality of trial reporting findings for force/time integral.

Ref.	Orthosis Design	Quality Index Score (%)	n =	Condition randomisation	Experienced runners	Acclimatization period
[60]	Custom-moulded soft: direct moulded	40	1	✗	✓	✓

## Conclusion

The studies included in this review are of low methodological quality, with the most confounding error being the lack of randomisation to the order of conditions. The most widely reported kinetic outcomes were loading rate and impact force, however the effect of foot orthoses on these variables remains unclear. In contrast, current evidence suggests that a reduction in the rearfoot inversion moment is the most consistent kinetic effect of foot orthoses during running.

This systematic review has evaluated the evidence surrounding the effects of foot orthoses on lower extremity kinetics during running. The findings demonstrate systematic effects that may inform the direction of future research in this field, as further evidence is required to define the mechanism of action of foot orthoses during running. Continuation of research in this field will enable targeting of design parameters towards biomechanical variables that are supported by evidence, and may lead to advancements in clinical efficacy.

## Competing interests

The authors declare that they have no competing interests.

## Authors' contributions

AM conceived the study design, conducted the systematic review, interpreted the findings and drafted the manuscript. CP reviewed the manuscript and provided academic support throughout.

## Author's information

AM is an Honours student within the Department of Podiatry, La Trobe University. CP is a Senior Lecturer within the Department of Podiatry, La Trobe University.
